# Temporal and spatial pattern analysis of escaped prescribed fires in California from 1991 to 2020

**DOI:** 10.1186/s42408-024-00342-3

**Published:** 2025-01-09

**Authors:** Shu Li, Janine A. Baijnath-Rodino, Robert A. York, Lenya N. Quinn-Davidson, Tirtha Banerjee

**Affiliations:** 1 Department of Civil and Environmental Engineering, University of California, Irvine, Irvine, CA USA; 2 Department of Environmental Science, Policy, and Management, University of California, Berkeley, Berkeley, CA USA; 3 University of California Agriculture and Natural Resources, Eureka, CA USA

**Keywords:** Escaped prescribed fires, Fuel treatment, Wildfire

## Abstract

**Background:**

Prescribed fires play a critical role in reducing the intensity and severity of future wildfires by systematically and widely consuming accumulated vegetation fuel. While the current probability of prescribed fire escape in the United States stands very low, their consequential impact, particularly the large wildfires they cause, raises substantial concerns. The most direct way of understanding this trade-off between wildfire risk reduction and prescribed fire escapes is to explore patterns in the historical prescribed fire records. This study investigates the spatiotemporal patterns of escaped prescribed fires in California from 1991 to 2020, offering insights for resource managers in developing effective forest management and fuel treatment strategies.

**Results:**

The results reveal that the months close to the beginning and end of the wildfire season, namely May, June, September, and November, have the highest frequency of escaped fires. Under similar environmental conditions, areas with more records of prescribed fire implementation tend to experience fewer escapes. The findings revealed the vegetation types most susceptible to escaped prescribed fires. Areas with tree cover ranging from 20 to 60% exhibited the highest incidence of escapes compared to shrubs and grasslands. Among all the environmental conditions analyzed, wind speed stands out as the predominant factor that affects the risk of prescribed fire escaping.

**Conclusions:**

These findings mark an initial step in identifying high-risk areas and periods for prescribed fire escapes. Understanding these patterns and the challenges of quantifying escape rates can inform more effective landscape management practices.

**Supplementary Information:**

The online version contains supplementary material available at 10.1186/s42408-024-00342-3.

## Resumen

**Antecedentes:**

Las quemas prescriptas juegan un rol crítico en la reducción de la intensidad y severidad de futuros incendios, haciéndolo mediante el consumo amplio y sistemático de la vegetación (biomasa) acumulada. Mientras que la probabilidad de escapes de quemas prescriptas en rodales y otros tipos de vegetación es muy baja, sus consecuentes impactos, particularmente cuando derivan en grandes incendios, causan una gran preocupación. La mejor manera de entender este intercambio entre la reducción del riesgo de incendio y el escape de quemas prescriptas es mediante la exploración de los patrones en los registros históricos de quemas prescriptas. Este estudio investigó los patrones espacio-temporales de escapes de fuegos de quemas prescriptas en California desde 1991 y 2020, ofreciendo indicios a los manejadores de recursos para que puedan desarrollar un manejo efectivo de los bosques y estrategias de tratamientos para la vegetación.

**Resultados:**

Los resultados revelan que los meses cercanos al comienzo o a la finalización de la estación de fuegos, en este caso mayo, junio, setiembre y noviembre, tienen la mayor frecuencia de escapes de fuegos. Bajo las mismas condiciones ambientales, áreas con mayores registros de implementación de quemas prescriptas, tienden a tener menos escapes de fuegos. Los resultados revelan los tipos de vegetación más susceptibles al escape de quemas. Áreas cuya cobertura forestal variaba entre 20% a 60% exhibieron la mayor incidencia de escapes de fuegos comparadas con arbustales o pastizales. Entre las condiciones ambientales analizadas, la velocidad del viento aparece como el factor predominante que afecta el riesgo de escape de una quema prescripta.

**Conclusiones:**

Estos hallazgos marcan un paso inicial en la identificación de áreas de alto riesgo y períodos de escape del fuego en quemas prescriptas. El entendimiento de estos patrones y los desafíos que implican la cuantificación de las tasas de escape pueden orientarnos sobre cómo implementar prácticas de manejo más efectivas.

## Introduction

The number of annual wildfires, the total burned area, and property damage have reached unprecedented levels in the past decades, reflecting a new wildfire regime (Dennison et al. 2014; Li and Banerjee 2021; Shuman et al. 2022). The change is caused by a combination of climate change, fuel accumulation, and forest densification due to fire exclusion, forest management practices, and insufficient fuel treatments that adequately replace the role frequent fire once had (Miller et al. 2020). Vegetation management is one of the primary mitigation measures that aim to proactively prepare the landscape for the inevitability of wildfires (Jazebi et al. 2019). Vegetation management can include a wide variety of activities, such as constructing fuel breaks, prescribing fires, and mechanical thinning (Prichard et al. 2020; Baijnath-Rodino et al. 2023; Banerjee et al. 2020; Banerjee 2020). Although mechanical thinning can be effective on its own if applied properly, prescribed (Rx) fire-alone or in conjunction with a mechanical treatment-is widely considered a relatively fast and highly effective treatment for reducing the severity of wildfires (Prichard et al. 2021; Ryan et al. 2013).

From an ecological standpoint, periodic low-intensity prescribed burning in fire-adapted forested landscapes (such as western coniferous forests) can reduce and maintain low amounts of surface and ladder fuels in forests (Keane 2008). They are frequently used in meadows, grasslands, and coniferous forests (Dyer 2002; Bradstock et al. 2006; Ryan et al. 2013; Engber et al. 2011), when conditions of weather and fuel moisture create safe burn conditions (Dether and Black 2006). These periods are called burn windows. Because they can occur infrequently and for a short duration during the course of a year, predicting their occurrence and being ready to conduct burns is pivotal in advancing the use of prescribed fire (Baijnath-Rodino et al. 2022; Striplin et al. 2020; Chiodi et al. 2019). Besides its effectiveness in reducing wildfire severity, prescribed burning also has the advantages of low economic cost and promoting fire-adapted flora (Finney et al. 2007; Faivre et al. 2016; Ryan et al. 2013). However, given the extensive backlog of untreated forests, it will take decades of fuel treatments carried out at faster rates and on larger spatial scales to have impacts at the ecosystem level (North et al. 2012; Kolden 2019). In 2020, the US Forest Service and the State of California announced a joint state-federal initiative to increase the annual scale of fuel vegetation treatment to one million acres (404,686 hectares) by 2025. This initiative involves expanding the use of prescribed fires as a key strategy and will be implemented by California state agencies such as CAL FIRE and other state entities, in partnership with the US Forest Service (Hazelhurst 2020).

Despite the necessity of prescribed fires to reduce the risk of wildfires in the western United States (Kolden 2019), their use has been limited in both speed and scale, due to several policy and operational barriers (Schultz et al. 2019; Ryan et al. 2013). A perceived obvious risk is the potential for escape, where ignitions occur outside the designated burn area during prescribed fires, becoming too large or difficult for on-site equipment and personnel to manage, thus necessitating external resources for suppression. Prescribed fire escapes are generally considered rare events; according to the 2022 National Prescribed Fire Program Review by the Forest Service Chief, the estimated escape rate is approximately 0.16% among the 4500 prescribed fires conducted annually across the United States by the Forest Service (USFS 2022a). However, even a small number of escapes possess the potential to escalate into large wildfires, posing significant threats to nearby communities and properties (Kobziar et al. 2015; Quinn-Davidson and Varner 2011). Escapes can lead to the suspension of prescribed fire programs across the country, increasing the backlog of needed treatments and increasing the difficulty of approving future burn plans (York et al. 2020). The Calf Canyon prescribed fire, for example, was conducted in January 2022 and subsequently reignited and escaped in April 2022. The Las Dispensas prescribed fire conducted on April 6, 2022, escaped and became the Hermits Peak wildfire and joined the Calf Canyon wildfire on April 22, resulting in one of the largest wildfires in New Mexico’s recorded history. The suppression of the 340,000-acre (137,600 hectares) Hermits Peak Fire cost approximately 100 million USD (USFS 2020). Almost simultaneously, another prescribed fire about 300 km south of the Hermits Peak Fire crossed the control line and escaped as the Overflow Fire. Despite being rapidly brought under control, approximately 1900 acres (769 hectares) were burned in total (NewMexicoFireInformation 2022). The US Forest Service then issued a “90-day prescribed fire review” on 22 May 2022, suspending all prescribed fire activities (USFS 2022b) pending further scrutiny. Escaped prescribed fires that result in moratoriums in their broad-scale application have the potential to defeat their intention-to reduce wildfire severity and ecosystem restoration, among others. Thus, understanding the historical trends in escaped prescribed fires is crucial for quantifying risks of escape events as well as strategically implementing future prescribed burns (Waldrop and Goodrick 2012).

Accurate quantification of the frequency and rates of prescribed fire escapes is restricted by limitations in monitoring data quality, particularly concerning prescribed fires that occur on private land, such as crop fires. Although the major large-scale escape events from prescribed fires on private land would be documented as wildfires in databases such as the Fire Perimeter database released by the California Department of Forestry and Fire Protection (CAL FIRE) (FRAP 2018), there is an evident gap in systematic recording for prescribed fires that do not escape on private land. This deficiency can lead to an overestimation of escape rates. In addition, while detailed reports on individual escape events provide valuable insight into local prescribed burn practices, their broader applicability is limited. There is an urgent need for a continuous and systematic analysis of temporal and spatial patterns as well as subsequent trends in prescribed fire escapes. However, existing statistics heavily rely on questionnaires and interviews (Miller et al. 2020; Weir et al. 2019), proving insufficient to establish a comprehensive quantitative understanding of the true extent of prescribed fire escapes.

To overcome the limitations of existing data we propose to conduct a meta-analysis encompassing diverse datasets and methodologies. By synthesizing data from multiple sources, including official fire datasets, government reports, and social sensing data, we aim to aggregate and analyze a comprehensive dataset that spans temporal and spatial scales. Leveraging a meta-analytic framework will allow for a better understanding of prescribed fire escape risks, providing valuable insights for resource managers and policymakers to enhance fire management strategies and mitigate potential impacts. The exploration of the indicators for potential escape events from prescribed fires will have significant importance to resource managers across different jurisdictions. To highlight the need for improved data and to provide new methods to quantify prescribed fire escape risk, we ask the following questions about prescribed fires using currently available data: (1) Are there seasonal or monthly trends of escaped prescribed fires in California? (2) What are the spatial characteristics of escaped prescribed fires? (3) Given that the ignition and spread of wildfires are collectively influenced by three influential dimensions-weather, topography, and fuel-which environmental factors are associated with prescribed fire escapes? This study has the potential to serve as a model for similar assessments, emphasizing the critical role of enhanced data quality and novel methodologies in understanding and mitigating prescribed fire escape risks.

## Material and methods

### Data

#### Escaped prescribed fires

The collection of prescribed fire data involved information from two databases: the California Department of Forestry and Fire Protection (CAL FIRE), including records dating back to the early 1900s, and the Monitoring Trends in Burn Severity (MTBS) dataset, including records from 1984 onward. Considering recent advancements in data recording integrity and systematic approaches, we selected the data spanning the preceding three decades, specifically from 1991 to 2020. All of these data sets provide comprehensive details, including the location of the fire, the ignition date, and the final burned areas. However, these databases mainly recorded fires that were planned and conducted by government or fire management agencies, with incomplete records of small prescribed fires conducted on private lands. After eliminating duplicate records, the total number of prescribed fire records in California from 1991 to 2020 was 4679.

Escaped prescribed fire records that do exist in California are mainly available from CAL FIRE. CAL FIRE has two programs that keep track of prescribed fire escapes: Fire and Resource Assessment Program (FRAP) (FRAP 2018) and California Incident Data and Statistics Program (CALSTATS). The Fire Perimeter project of FRAP collects fire perimeter data from the Bureau of Land Management (BLM), CAL FIRE, National Park Service (NPS), and USFS and builds an ESRI ArcGIS file geodatabase. The fire history dates back to 1954 and includes 19 distinct wildfire causes, each classified by a specific code ranging from 1 to 19. Among these, cause code 18 denotes “escaped prescribed fires.” CALSTATS collects fire records throughout the state of California using the National Fire Incident Reporting System (NFIRS) (CALSTATS 2021). Records can be obtained in the format of spreadsheets by submitting a request, which includes information on the types, causes, and locations of fires. We requested the fire records with NFIRS Incident Type codes 140–143, which means wildland fires, and 561, which means unauthorized burning. Then, the escaped prescribed fires were filtered by NFIRS Code 75, that is, agricultural or land management burns, including prescribed burns. As the primary focus of this study is to explore patterns related to escaped prescribed fires, records of prescribed fires carried out on agricultural lands were retained, considering the potential risk of their escape leading to large fires in wildland areas. During the time period of 1991 to 2020, there were 74 escaped prescribed fire records in the FRAP database and 239 records in the CALSTATS database, including 3 recorded escapes that are duplicates. Analyses conducted on the dataset excluding agricultural fires can be found in the Supplementary Information (SI) (8. Results - Spatiotemporal Patterns excluding agricultural fires).

Furthermore, with the increasing presence of social media, social sensing data have also become an important source of recording and complementing data outside the official databases. Wildfire Today is a website that collects and releases wildfire news in real time (Wildfire Today 2024). The data from Wildfire Today supplement and validate the official fire datasets. Their information is compiled from government reports, social media sources, guest writer submissions, and fire monitoring dashboards. For example, the escaped fire recorded in Victorville, California, on March 31, 2015, was not found in either of CALFIRE’s databases. In addition, their records of independent fire events have become increasingly comprehensive and complete in recent years. We collected the escaped prescribed fire news and reports from the Wildfire Today archives as an external data source. There are 13 records that point to actual escaped prescribed fires from 2008, the year the website was developed, to 2020.

To organize the data from different databases, we extracted the fire start date, fire name, and location information from all three databases, identified the data source, and sorted them together according to the fire start date. After removing duplicates, there are 310 escaped prescribed fire records with accurate locations, represented by coordinates of the centroid point of fire perimeters, in California from 1991 to 2020, about 10 escaped per year.

#### Environmental variables

The climate and topography data were extracted mainly from the spatial climate dataset Parameter-Elevation Regressions on Independent Slopes Model (PRISM) (PRISM 2020). These environmental variables were selected based on parameters relevant to determining the burn window for prescribed fires, specifically wind speed, maximum vapor pressure deficit (VPD), maximum temperature, and precipitation, which contribute to escape events (Baijnath-Rodino et al. 2022). Multi-year (2007–2013) Annual Average Wind Speed in meters per second, at 10 m above surface level, was extracted from the Wind Integration National Dataset (WIND) Toolkit, developed by National Renewable Energy Laboratory. The resolution above California is 2 km $\times$ 2 km (Draxl et al. 2015). The maps for environmental variables can refer to SI (1. Data - Environmental Variables).

The Landscape Fire and Resource Management Planning Tools program uses “plot-level ground-based visual assessments and Lidar observations,” providing information about the percentage of canopy cover of herbaceous plants, shrubs, and trees (LANDFIRE 2016). To investigate which types of vegetation are related to the highest escape risk, the Fuel Vegetation Cover (FVC) (SI Fig. S1(g)) of the program with a resolution of 30 m was overlayed on the escape record to calculate the spatial correlation. Additionally, the National Vegetation Classification (NVC) (SI Fig. S1(h) with the same resolution was used to further determine the dominant plant species in the prescribed fires that escape.

### Methods

#### Temporal analysis

To analyze the temporal variation of the escaped prescribed fires, the total annual, seasonal, and monthly counts and the burned area were plotted against time. This study adopts the meteorological seasons used in California, with December, January, and February (DJF) representing winter; March, April, and May (MAM) representing spring; June, July, and August (JJA) representing summer; and September, October, and November (SON) representing fall. Escaped prescribed fires with burned areas exceeding 5000 acres (2023 hectares) were considered extreme events, as they could significantly skew the results of burned area analyses by creating notable peaks in the months and seasons when they occurred. Therefore, these extreme events were treated as outliers, plotted as individual points in the figures, and excluded from the temporal analysis of total burned area. Statistics that include these extreme events are available in SI (2. Methods - Outliers Detection and Treatment).

The direct calculation of the probability of prescribed fire escape over the last 30 years, using escaped records divided by total prescribed fires, is problematic due to differences in data sources. Specifically, not all prescribed fires have records indicating whether they escaped, and some escaped events may not have been recorded in the database. This discrepancy has a more significant impact on the burned area than on fire counts, especially when considering extremely large escaped fires (outliers). To address this issue and take total prescribed fire counts into account when analyzing the temporal patterns of escaped prescribed fires, Bayesian hierarchical models were used to investigate the occurrence probability and mean occurrence counts on a monthly basis. Specifically, the binomial distribution is apt for characterizing the count of successes in a sample drawn from a population, and the Poisson distribution is applied in describing the probability of a particular number of events taking place within a fixed interval of time or space. Therefore, we opted for the Binomial-Beta model (Wilcox 1981) and the Poisson-Gamma model (Foster and Bravington 2013) to independently estimate the occurrence probability and occurrence counts of escaped prescribed fires each month. The detailed description and equations can be found in SI (3. Methods - Bayesian Models).

#### Spatial analysis

The analysis of spatial patterns was initiated with a comprehensive spatial randomness test (CSR) designed to delineate the first-order property of point processes Wiegand and A. Moloney (2004). This test measures the spatial randomness of escaped prescribed fires, and the $\chi ^2$ (chi-squared) statistic serves as a metric of the variation between observed and expected point distributions in the absence of any relationship between them in this test. The second-order property of point processes, based on pairs of points, is used to characterize how the spatial point pattern deviates from complete spatial randomness (cluster or repulsion). The G-function, K-function, and L-function (Ripley 1976, 1977; Diggle 2003) are three common tools to measure how the spatial point pattern deviates from a homogeneous Poisson distribution, and their equations are detailed in SI (4. Methods - Complete Spatial Randomness Test; 5. Methods - Identification of Clustering or Repulsion Patterns). In general, if the estimated functions based on escaped prescribed fire records (observations) consistently exceed the theoretical distribution, it indicates that the observed point patterns contain more points than expected under the theoretical homogeneous Poisson distribution. This suggests that the spatial distribution of escaped prescribed fires is clustered. The confidence intervals of the theoretical Poisson distributions for the G, K, and L functions were calculated by Monte Carlo simulation (Genton et al. 2006; Turner 2009) with 99 iterations, to avoid randomness in the single theoretical distribution estimation. As long as the observed distribution falls within the envelope of the confidence interval, it is completely spatially random.

As a reference for interpreting the spatial patterns of escape events, the spatial intensity function of the occurrence of escape events, which describes the distribution of escape events density, was estimated by non-parametric kernel estimates (Kuter et al. 2011; Liu et al. 2010). The choice of KDE is made because this method only looks at the point patterns themselves, without covariables; besides, it is non-parametric which means it does not need to include the model of the underlying process. Here, the mean squared error (MSE) between the kernel estimator and the actual counts was used to select the bandwidth, for which the optimal bandwidth should minimize the MSE, and the optimal value is 0.17.

To establish the relationship between environmental variables and escaped prescribed fire incidents, logistic regression analysis was employed. As a form of generalized linear regression, logistic regression facilitates the dichotomization of dependent variables based on multiple independent attributes (Hosmer Jr. et al. 2013; De Vasconcelos et al. 2001). Environmental conditions corresponding to each escaped prescribed fire point were extracted and incorporated into the logistic regression model. Subsequently, through the model training process, the weights of individual variables were determined, indicating the influence of various meteorological and topographic factors on the occurrence of escape.

Spatial point clusters were analyzed in ArcGIS Pro using three methods: density-based clustering, hot spot analysis, and multivariate clustering. Density-based clustering aims to identify densely concentrated points distinguished from lower-density or vacant regions. Applying the Ordering Points to Identify Clustering Structure (OPTICS) technique (Agrawal et al. 2016), this method detects clusters considering the spatial distribution of points and their distances to a specific number of neighbors. The minimum feature in each cluster was set to 4.

The Hot Spot Analysis tool calculates the Getis-Ord Gi* statistic for each feature in a data set to determine whether local patterns exhibit significant deviations from global features (Getis and Ord 1992; Ord and Getis 1995). Results from this analysis provided *z*-scores and *p*-values, which served as indicators of spatial clustering of either high or low values. Higher *z*-scores that are statistically significant denote intensified clustering of high values (hot spots), whereas lower statistically significant negative *z*-scores suggest notable clustering of low values (cold spots).

The multivariate clustering method applied the K-means algorithm in an attempt to identify clusters characterized by high intra-cluster similarity and significant inter-cluster differences (Jian 2009; Hinde et al. 2007). The selection of cluster numbers was attempted with 3, 4, and 5 clusters, ultimately determining 4 clusters as optimal due to its effective separation of clusters with distinct characteristics, all while maintaining minimal complexity.

The details and equations of above analyses were provided in SI (6. Methods - Kernel Density Estimation).

## Results

### Temporal patterns

**Fig. 1:**
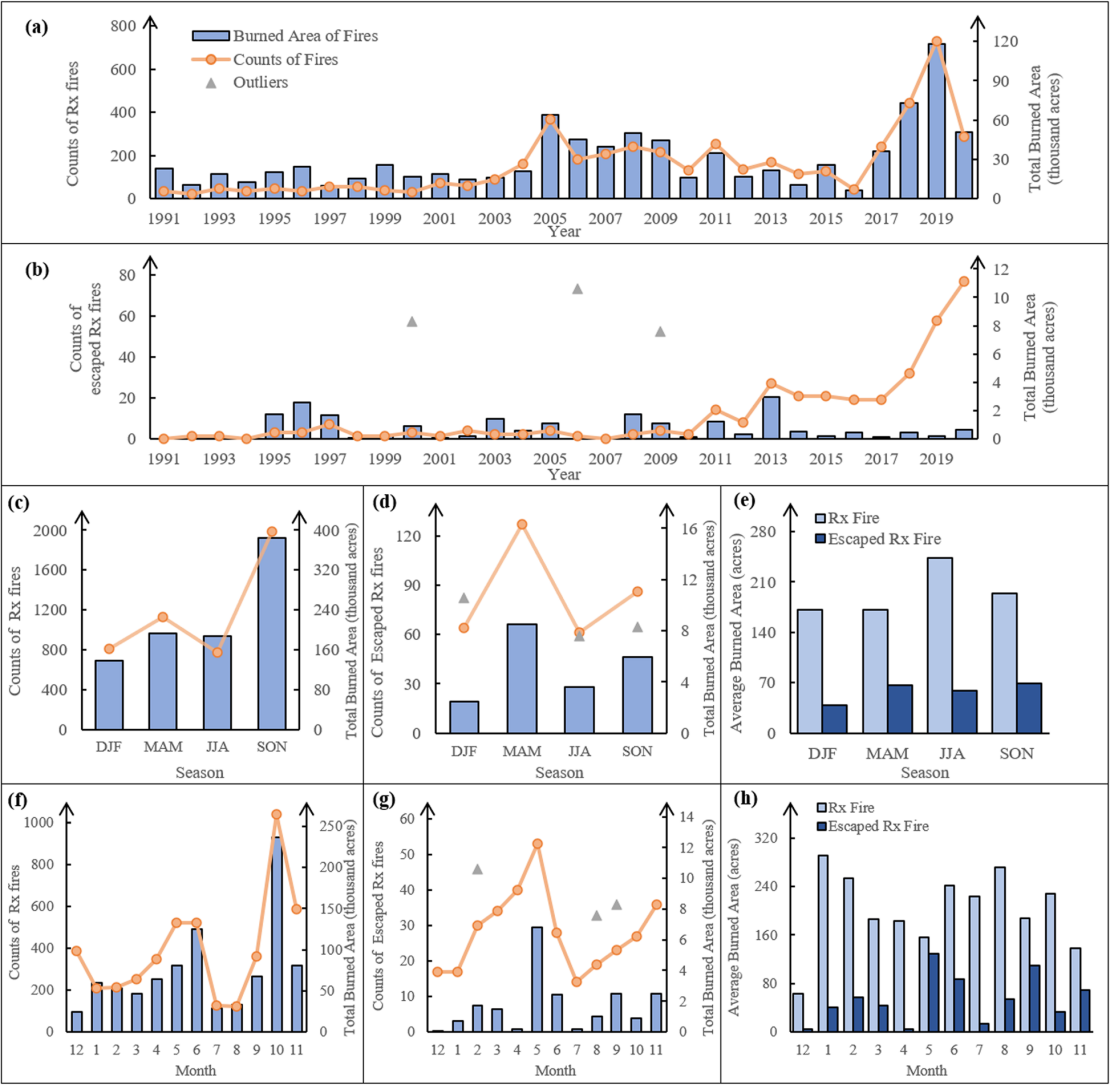
Temporal patterns of Rx fires and escaped Rx fires in California from 1991 to 2020. Temporal patterns are depicted on the basis of yearly (**a**, **b**), seasonal (**c**–**e**), and monthly (**f**–**h**) occurrences. The seasons are denoted by the months they contain, with DJF representing winter, MAM representing spring, JJA representing summer, and SON representing autumn. Panels (**a**, **c**, and **f**) present data for Rx fires, while panels (**b**, **d**, and **g**) represent escaped Rx fires. Panels (**e** and **h**) represent the average burned area for a single Rx fire and a single escaped Rx fire in each season (**e**) and each month (**h**). The orange points and lines correspond to fire counts, the blue columns indicate the total burned area of the fires, and the grey triangles represent the outliers in escaped prescribed fires with burned areas larger than 5000 acres (2023 hectares)

Due to limitations in the existing prescribed fire datasets, accurately determining the proportion of escapes in prescribed fires is a challenge. Consequently, Fig. 1 shows the side-by-side comparison of the 30-year temporal patterns of annual, seasonal, and monthly counts of prescribed fires and escaped prescribed fires, along with their associated burned areas from 1991 to 2020.

The annual trends of both prescribed fires and escaped prescribed fires were significantly influenced by the consistency of the record sizes. In 1991, only 36 prescribed fires were recorded, covering a total burned area of 22,810 acres (9230 hectares). This number rose considerably in 2005, with 369 prescribed fires recorded, resulting in a total burned area of 64,240 acres (26,000 hectares). In 2019, the number of prescribed fires surpassed 700, covering more than 100,000 acres of burned area (Fig. 1a). Over the past three decades, 52.58% of escaped prescribed fires (163 out of 310) occurred in cropland. The risk of escape for prescribed fires related to agricultural use is greater than that for forest management, as only 11.35% of prescribed fires (531 out of 4,679) were conducted in cropland.

The triangular markers in Fig.1b denote three escaped prescribed fires characterized by exceptionally large burned areas ($\ge$ 5000 acres, or 2 hectares): the Weinstein Fire in 2000 (8284 acres or 3352 hectares), the Sierra Fire in 2006 (10,592 acres or 4286 hectares), and the Big Meadow Fire in 2009 (7553 acres or 3056 hectares). The transition of prescribed fires into extreme wildfires is influenced by a combination of environmental factors, fire management practices, and occasional human actions. While these extremely large escaped prescribed fires are influenced by the same environmental factors as smaller escaped fires, their significant burned areas would heavily skew the results of the temporal analysis. We chose not to exclude them entirely, as these large events are often the ones that bring public attention to prescribed fire escapes. Instead, we treated them separately, excluding them from the general statistical analysis while representing them as individual markers to highlight their significance (Fig. 1d, g).

Prescribed fires exhibit a prominent peak during autumn (SON) in both counts and total burned area (Fig. 1c). Looking at individual months, October emerges as the month with most implementation of prescribed fires (Fig. 1f). Moreover, May, June, and November also experience intensive prescribed fire implementations ($\ge$ 500 fires), with prescribed fire counts of 522, 519, and 587, respectively. The average burned area of a single prescribed fire is the largest during the summer (JJA) (Fig. 1e). Apart from the summer period, January, February, and October notably have larger average burned areas ($\ge$ 200 acres or 81 hectares) compared to other months (Fig. 1h). Although the implementation of prescribed fires may be less frequent during January and February, the scale of each implementation is substantial.

During the past 30 years, escaped prescribed fires have predominantly occurred during spring (MAM), with May experiencing the largest total burned area if outliers are disregarded (Fig. 1d, g). From January to May, the count of escape records steadily increases, while the total burned area remains relatively stable, except for a notable increase in May. As June arrives, both the total counts and burned areas of escaped fires start to decline due to the beginning of the wildfire season and the adoption of more cautious prescribed fire implementation. However, from July onward, the total counts of escapes show a second sustained upward trend, with relatively higher burned areas observed in September and November. Notably, the average single escaped fire burned area is significantly high in May and September, followed by June and November, corresponding to months close to the beginning and end of the wildfire seasons.

Temporal statistics that consider only fires in natural vegetation, excluding agricultural burns are provided in SI (Fig. S5). The exclusion of agricultural fires from this analysis does not significantly affect the annual, seasonal, or monthly temporal patterns observed for both prescribed fires and escaped prescribed fires. The sole notable change is a significant increase in the average burned area of escaped prescribed fires occurring in May, June, and September.

**Fig. 2:**
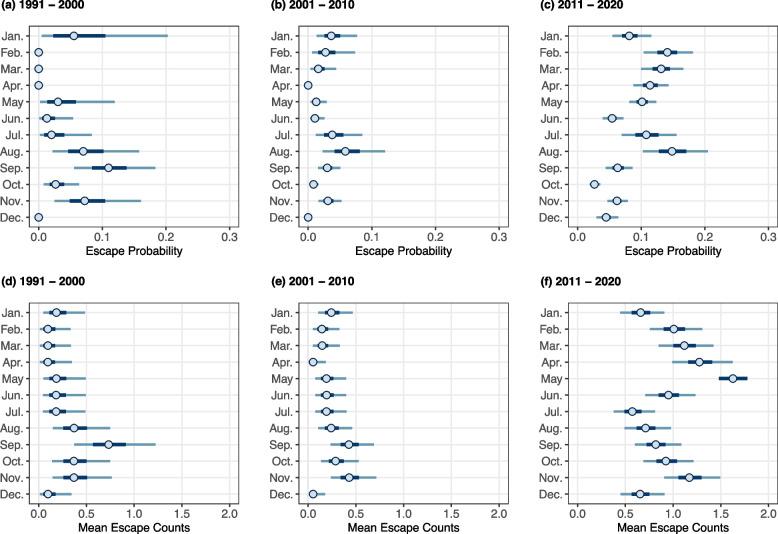
Distribution of escaped Rx fire occurrence probability and mean counts in each month across California from 1991 to 2020. The probability distribution was estimated using the Binomial-Beta model, while the mean occurrence count distribution was estimated using the Poisson-Gamma model. The points represent the mean value, the thick inner segments represent 50% posterior credible interval and the thinner outer segments represent 90% posterior credible interval

The occurrence probability of escaped fires in each month over the last three decades by decade was analyzed using the Binomial-Beta Bayesian model (Fig. 2d–f). The points represent the estimated mean escape probability in each month. The thicker line represents the 50% posterior credible interval and the thinner outer segments represent 90% posterior credible interval, indicating that, given the observed historical data, there is a 50% and 90% probability that the true estimate lies within the interval, respectively. The credible intervals of monthly escape probability from 1991 to 2000 are wide, indicating high uncertainty, owing to limited data and inconsistent entry frequency and quality. Since 2001, the credible intervals have become narrower, indicating an improvement in the data integrity of escaped prescribed fire records.

During the period of 2001–2010, the highest escape probability occurs in July and August, with considerable uncertainty, as these months fall within the wildfire season. This pattern remains consistent from 2011 to 2020. From 2001–2010 to 2011–2020, the probability of escape occurrence increased in all months while maintaining similar monthly trends. The most significant increases are observed in late winter and spring, particularly in February, which even becomes a minor peak of escape probability. After that, the probability of escape gradually decreases until June. The mean escape probability in autumn and winter remains lower than 0.1. While the month with the highest escape probability may not coincide with the month having the most escape records or the largest escaped fire burned areas, the conclusion that the risk of escape is higher just before the wildfire season compared to right after the wildfire season remains valid. Given that fall burns are executed most frequently (Baijnath-Rodino et al. 2022), selecting suitable burn windows within this period becomes essential to mitigate the risk of escapes.

Meanwhile, the mean count of possible escapes in each month for the past 30 years by decades was analyzed by the Poisson-Gamma model (Fig. 2d–f). From the first decade to the last, it is evident that mean counts increase in all months. The monthly distribution of the mean fire count in the last decade (Fig. 2f) closely aligns with the 30-year total counts of escaped prescribed fires by month (Fig. 1g), confirming the rapid increase in escaped fire records between 2011 and 2020. During this period, a new peak emerged in the spring, in addition to the existing autumn peak, which is consistent with the 30-year seasonal distribution shown in Fig. 1d. This indicates a significant increase in spring escapes from 2011 to 2020, making it a major peak by the end of the 30-year period.

### Spatial patterns

The results of the Complete Spatial Randomness Test (CSR) for escaped prescribed fires revealed a *p*-value of $10^{-4}$, indicating that their spatial intensity (i.e., the density) is significantly non-constant at a significance level of 0.05. This suggests that the escapes are not randomly distributed. The estimation of G, k, L functions (refer to SI Fig. S4) suggests that the spatial distribution of the escaped prescribed fires is clustered.

**Fig. 3:**
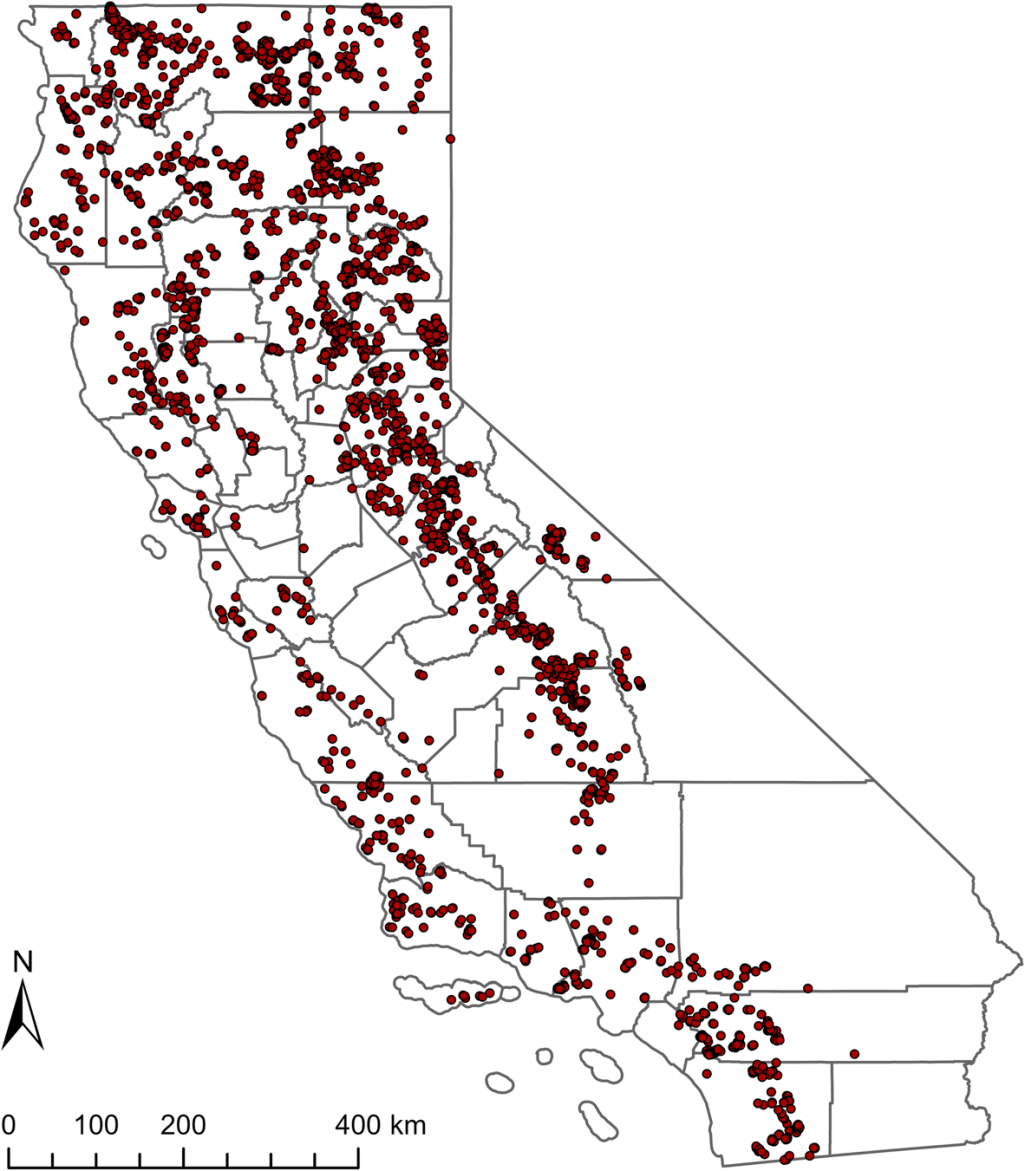
Spatial distribution of escaped prescribed fires in California from 1991 to 2020. Red points represent the locations of escaped prescribed fires, while county boundaries are shown in grey

The spatial distribution of escaped prescribed fires can be viewed as a point process (Fig. 3). Evidently, the escapes were concentrated in northern California and central California, particularly along the Sierra Nevada mountains and in the Central Valley. Additionally, there were notable hot spots of escapes in the southwestern corner of California, spreading across the San Bernardino National Forest and the Angeles National Forest on the San Gabriel Mountains.

**Fig. 4:**
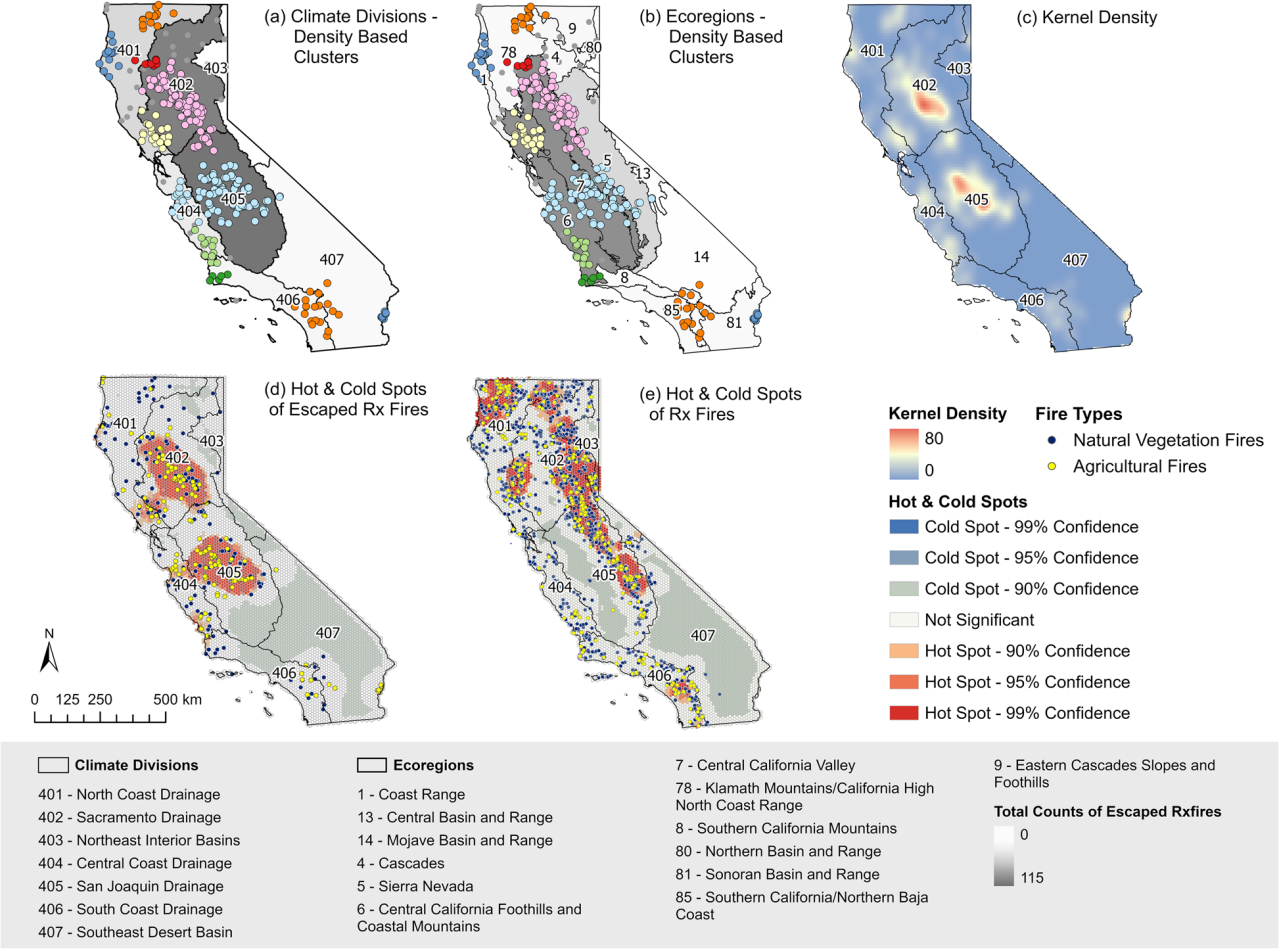
Spatial distribution of escaped Rx fire across California. The points in (**a** and **b**) represent escaped prescribed fire locations, with different colors representing distinct clusters. A total of 10 clusters were identified, and clusters with the same color, but not adjacent and located far apart, are considered separate clusters. Varying levels of grey in panels (**a**) for climate divisions and (**b**) for ecoregions denote the magnitude of total escaped prescribed fire counts. Panel (**c**) depicts the estimation of kernel density for escaped Rx fires; panel (**d** and **e**) depict the identification of hot and cold spots for Escaped Rx fires and Rx fires, respectively. The points indicate the locations of fires, with different colors representing the types of prescribed fires initiated

To determine the high-density regions for escaped prescribed fires, we classified their clusters based on the distances among individual escape events and their associated density (Fig. 4). The cluster map was overlaid with California’s climate divisions and ecoregions to identify the climate and ecosystem characteristics associated with each cluster (Fig. 4a, b). Considering the climate divisions, the two largest clusters were situated in the middle of the Sacramento Drainage (climate division 402) and San Joaquin Drainage (climate division 405) basin area. Additionally, three climate divisions along the coast, namely the North Coast Drainage (climate division 401), the Central Coast Drainage (climate division 404), and the South Coast Drainage (climate division 406), included the majority of the remaining escaped prescribed fires, exhibiting a moderate cluster density. Notably, the Southeast Desert Basin contains the only isolated cluster, located in the Palo Verde Valley and near the Colorado River Indian Reservation. In the analysis of ecoregions (Fig. 4b), it was found that the majority of the escaped prescribed fires classified into six clusters were concentrated in the Central California Foothills and Coastal Mountains (region 6) as well as the Central California Valley (region 7). Furthermore, the Sierra Nevada Mountains (region 5) contained points on the eastern periphery of the two largest clusters. The integrated density distribution of escaped prescribed fires at the state level (Fig. 4c) shows consistent spatial patterns with Fig. 4 a, b.

Due to the lack of an exact match between the prescribed fire database and the escaped prescribed fire database, calculating the rate of escape in a specific region may introduce some bias. To account for the overall prescribed fire implementation, a comparison was made between the hot and cold spots for prescribed fires and escaped prescribed fires across California (Fig. 4d, e). Hot and cold spots on the maps indicate whether the local point pattern is statistically different from the global features, i.e., it displays more intensity or sparsity. The initiation of prescribed fires in natural vegetation or agricultural lands were represented using different colors of points. Both maps reveal extensive areas of high-confidence hot spots, while the cold spots only appeared at a 90% confidence level, predominantly concentrated in the desert basin. The hot spots for prescribed fire records are concentrated in several regions, including the Northern Coast Drainage in northern California, the eastern area of the Sierra Nevada Mountains in central California, and a small cluster in the South Coast Drainage in Southern California. On the other hand, the hot spots for escaped prescribed fires exhibit patterns similar to the spatial density distribution, with concentrations observed in the Sacramento Drainage (climate division 402) and the San Joaquin Drainage (climate division 405), respectively.

The overlap of hot spots in the two maps is only evident in central California, particularly on the western side of the Sierra Nevada Mountains (Fig. 4d, e). Within the Sacramento Drainage, the overlaps largely coincide with the areas of highest density for escaped prescribed fires. However, in the San Joaquin Drainage, the overlaps occur on the eastern side of the high-density center for escaped prescribed fires, avoiding the main high-density area and containing only a moderate density of escaped prescribed fires. Consequently, the middle of the Sacramento Drainage emerges as one of the most common areas for prescribed burns as well as one of the areas with the highest occurrence of escape events. In contrast, the middle of the San Joaquin Drainage, where prescribed fires are more frequent, displays a moderate density of escapes. The highest density of escapes in the San Joaquin Drainage is situated to the west of the prescribed fire hot spot and is not contained within the prescribed fire hot spots at all. This pattern of prescribed fire and escaped prescribed fire hot spots adjacent to each other but not intersecting is consistent throughout the rest of California.

The spatial patterns of fires occurring mainly in natural vegetation, excluding agricultural burns are presented in SI (Fig. S6). Following the exclusion of prescribed and escaped agricultural fires, the distribution of hot spots for prescribed or escaped natural vegetation fires essentially remained unchanged. The primary hot spots continue to be located along the Central Valley, while additional relative hot spots emerged in northern California, areas noted for the high density of national forests.

### Environmental condition profile in escaped prescribed fire sites

Prescribed fires are often carried out in areas with high risks of wildfires. Despite the strict and careful selection of the date and area for the implementation of prescribed fires from the planning to execution phases, complete prevention of escapes remains a challenge (Ryan et al. 2013). Among the various factors contributing to escapes, environmental conditions within the burned area by prescribed fires pose the greatest difficulty in accurate prediction during planning or manipulation during implementation (Agastra 2022). Results from logistic regression, which examined all prescribed fires and escaped incidents throughout California as presented in Table 1, highlight wind speed as the predominant factor influencing the risk of prescribed fire escape. Specifically, higher wind speeds at 10 meters are associated with an increased risk of escape fires. Additionally, fuel vegetation cover is identified as a significant variable affecting escape risk. In contrast, other variables show limited influence on escape occurrences. The significant impact of wind, contrasted with the minor influence of other variables on escape occurrences, may be attributed to the high variability in wind speed and direction (Simpson et al. 2013; Sharples et al. 2012) and large uncertainty (Sanjuan et al. 2014) in wind prediction during prescribed fire practices. Additionally, California’s extensive spatial scale and diverse environmental conditions tend to diminish the effects of other environmental factors when analyzing escapes at the state level.

**Table 1:** Logistic regression results for explanatory environmental variables associated with escaped prescribed fires in California from 1991 to 2020

Variables	Coefficient	S.E.	*p*-value
Precipitation (in.)	− 0.0003	0.0002	< 0.05
Max temperature (°C)	− 0.0534	0.0730	0.46
Max vapor pressure deficit (hPa)	0.0390	0.0351	0.27
Fuel vegetation cover (%)	− 0.0227	0.0032	< 0.05
Wind speed (m/s)	0.1572	0.0685	< 0.05
Elevation (km)	− 0.0020	0.0002	< 0.05
Aspect (°)	− 0.0012	0.0006	0.06
Slope (°)	− 0.0189	0.0092	< 0.05

**Fig. 5:**
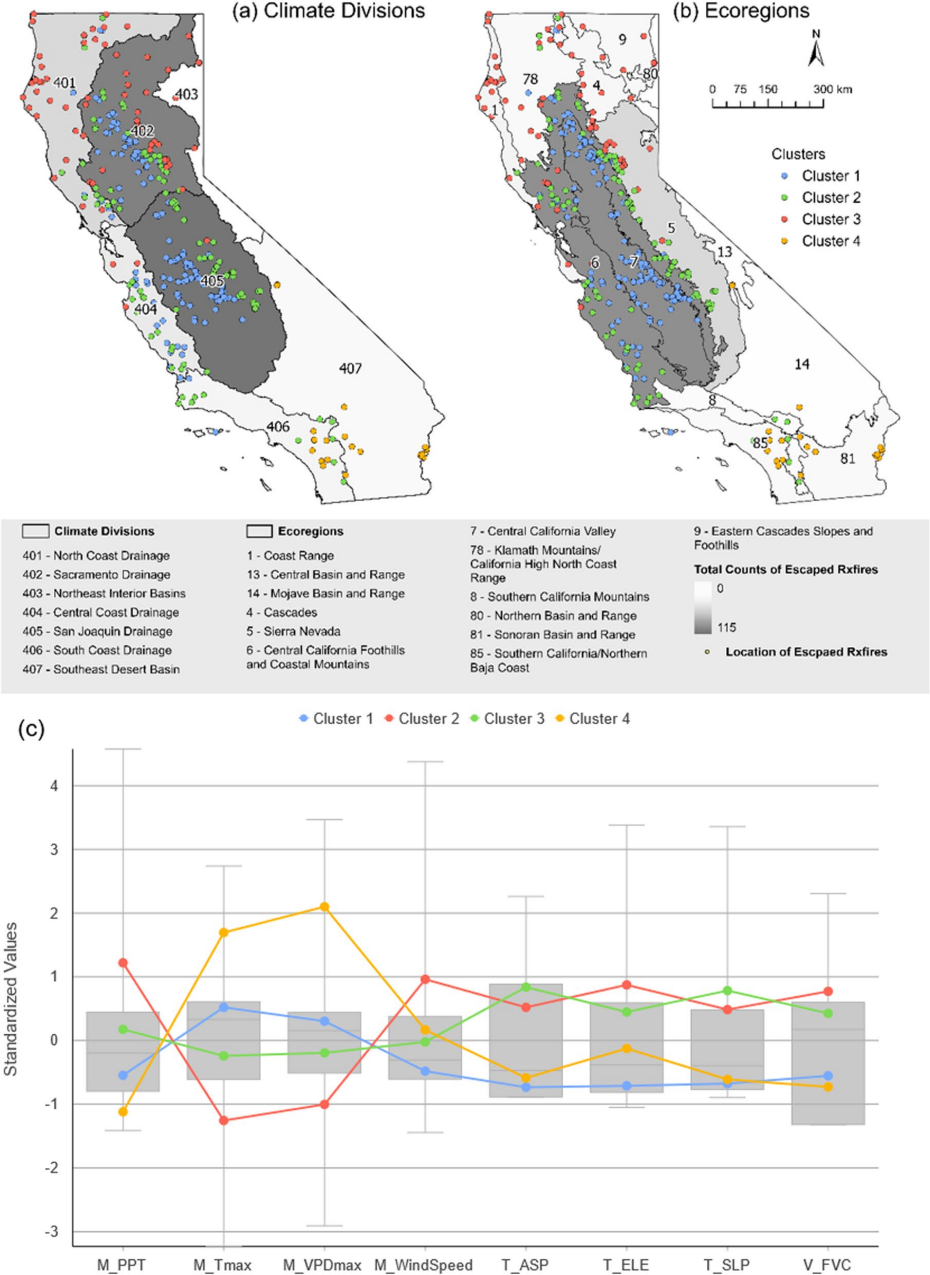
Multivariate clustering of escaped prescribed fires and total counts in environmental divisions. The points in panels (**a** and **b**) represent escaped prescribed fire locations, with different colors representing distinct clusters. A total of 4 clusters were classified using contributed environmental variables, namely precipitation (PPT), maximum temperature (Tmax), maximum vapor pressure deficit (VPDmax), mean wind speed at 10 m (WindSpeed), aspect (ASP), elevation (ELE), slope (SLP), and fuel vegetation cover (FVC). Varying levels of grey in panels (**a**) for climate divisions and (**b**) for ecoregions denote the magnitude of total escaped prescribed fire counts. Panel **c** shows the distribution of standardized environmental variables among multivariate escaped Rx fire clusters

Given the vast geographical expanse and complex climatic conditions of California, the causes of prescribed fire escapes exhibit considerable variation across spatial distributions. Therefore, the escaped prescribed fires were classified into four clusters based on the selected environmental variables (Fig. 5). This classification ensures that fires within the same cluster share the greatest similarities, while those in different clusters display the most significant variations. Upon overlaying climate division and ecoregion boundaries, it becomes obvious that the majority of cluster boundaries align with California’s ecoregions.

Cluster 1 mainly encompasses escaped prescribed fires within the Central California Valley (region 7), while also including scattered events within the Central California Foothills and Coastal Mountains (region 6). The Central California Valley is crucial for California’s agricultural production, featuring flat terrain, fertile soils, and a favorable climate, with nearly 70 percent of its land in cultivation (Kuminoff et al. 2000). Notably, escaped events in cluster 1 exhibit relatively low precipitation and wind speed, coupled with high maximum temperatures and vapor pressure deficits compared to average climatic conditions. The cluster demonstrates characteristics of lower elevation, aspect, and slope, suggesting the presence of flatter, lower terrains. The hot and dry summers in this region are deemed risky for the implementation of prescribed fires, and any instances of escape could have significant negative impacts on local agriculture.

Escaped prescribed fires within cluster 2 are primarily distributed along the ecoregion of Central California Foothills and Coastal Mountains, and Sierra Nevada, almost encircling cluster 1. This region is characterized by a Mediterranean climate, with hot, dry summers and cool, moist winters, and is predominantly covered by chaparral and oak woodlands. Grasslands are found at lower elevations, while patches of pine occur at higher elevations (Griffith et al. 2016). The climate conditions in cluster 2 are milder in comparison to cluster 1. This is evident through the presence of more precipitation, lower temperatures, lower vapor pressure deficits, and higher terrain. Prevailing afternoon winds and high vegetation cover in this region contribute to the occurrence and spread of the escapes.

Cluster 3 includes escaped fires dispersed throughout northern California, primarily located in the outer and upper regions of cluster 2. These fires span four main ecoregions: the Coast Range, Cascades, Klamath Mountains, and Northern Basin and Range. All of these regions are characterized by dense forests and rich biodiversity, shaped by complex geological formations of volcanic, granitic, and sedimentary rocks. While the Cascades and Klamath Mountains have a more temperate and moist climate, the Eastern Cascades experience greater temperature extremes (Griffith et al. 2016). Climate and topographical conditions in this cluster show similarities with cluster 2 but exhibit a more extreme nature. During the prescribed fire burn window, extreme caution is required due to the potentially high wind speed, low air humidity and dense vegetation cover (Fig. 5c).

Cluster 4 contrasts with the previous clusters by focusing on escaped prescribed fires that occurred in southern California, with occurrences distributed similarly between the South Coast and Southeastern Desert regions. While escaped fires in the South Coast region were found in areas of high vegetation density, all escaped fires in the Southeastern Desert region were exclusively associated with agricultural land use, particularly crops, as prescribed fires are typically not initiated in natural desert habitats. Both regions share common characteristics, including limited vegetation cover, a hot and arid climate year-round, and high wind speeds (Fig. 5c), which create significant challenges for the management and control of prescribed fires.

The same logistic regression procedure was applied to escaped natural vegetation fires, excluding agricultural fires resulting in coefficients for each environmental variable that remained unchanged. However, cluster classification was not conducted for escaped natural vegetation fires due to insufficient data size, which would not yield meaningful results.

Furthermore, the assessment of vegetation cover in escaped prescribed fires can be extended to specific vegetation types and land uses. As shown in Table 2, trees are the most prevalent vegetation type in areas of escaped fires, followed by herbs, with shrubs being the least common. Most escapes occurred in areas with 20–60% tree cover, while herb cover between 40 and 50% was also notably dominant. Within the National Vegetation Classifications, escapes were most frequently observed in California Montane Conifer and California Broadleaf forests. Among herb types, California Ruderal Grassland and Meadow were prominent, while California Xeric Chaparral was a common shrub type in escaped prescribed fires.

**Table 2:** Dominant vegetation classes and land use types in escaped prescribed fires

Vegetation type	Percentage (%)	Accumulated percentage (%)
**Fuel vegetation cover**		
Developed - roads	15.48	15.48
40 $\le$ tree cover $< 50\%$	11.31	26.79
30 $\le$ tree cover $< 40\%$	6.85	33.63
40 $\le$ herb cover $< 50\%$	5.95	39.58
50 $\le$ tree cover $< 60\%$	5.36	44.94
20 $\le$ tree cover $< 30\%$	5.06	50.00
**National vegetation classification**		
Developed - roads	15.48	15.48
California Montane Conifer Forest and Woodland	8.63	24.11
Western Warm Temperate Orchard	8.63	32.74
California Xeric Chaparral	7.14	39.88
California Broadleaf Forest and Woodland	6.85	46.73
California Ruderal Grassland and Meadow	5.95	52.68

The table displays the highest-ranking vegetation species until the cumulative percentage reaches 50%

Roads were identified as the most frequently impacted land use type associated with escaped prescribed fires, primarily due to their high density in agricultural regions. The agricultural area classified as Western Warm Temperate Orchard ranked third among the vegetation types affected by these escapes. These findings suggest that a significant proportion of escaped prescribed fires originates from agricultural and land management burns, as evidenced by the high incidence of these events occurring near roads. A table detailing escaped prescribed fires, excluding agricultural fires, is available in SI (8. Results - Spatiotemproal Patterns excluding agricultural fires). After excluding agricultural fires, the escaped natural vegetation fires predominantly occurred in areas with moderate tree, shrub, and herb density. While the dominant vegetation types in the escaped natural fires remained consistent with all escaped fires, the land use types of roads and orchards did not appear in the top 50% of common land use types associated with these escapes.

## Discussions

Although the temporal statistics demonstrate a general increase in the implementation of prescribed fires in California, especially from 2010 to 2020 (Fig. 1b), it is essential to consider the role of improved data collection standards and improved fire data system management, which has contributed to more accurate and comprehensive prescribed fire records. In the past, data on escapes of prescribed fires on private land were challenging to collect, and small-scale, controllable escapes were often unreported and undocumented (Weir et al. 2019). Consequently, only escapes that turned into large wildfires, and under the management of CAL FIRE’s units or cooperating agencies were included in the database, under the classification of wildland fires. Since 2014, the number of escaped prescribed fires has continued to grow, while the total burned area has stabilized-a trend also noted in other studies (Miller et al. 2020; Cummins et al. 2023). This pattern may reflect advancements in technology and improved control measures in the implementation of prescribed fires.

It is also notable that over the past three decades, almost half of escaped prescribed fires (163 out of 310) were initiated by crop fires. This statistic highlights the increased risk associated with prescribed fires conducted in agricultural settings. The unique features of croplands, such as the presence of highly combustible materials and their proximity to roads and infrastructure, contribute to the likelihood of fire escapes and fatalities (Twidwell et al. 2015). Initiating a prescribed fire on private land is often less strict than on public land due to differences in governance, resources, and liability concerns. Agricultural burn practices, in particular, may not always follow the strict protocols required for public land prescribed fires, potentially leading to less controlled conditions (Wilkin et al. 2024). In the U.S., private landowners must typically obtain permits and comply with local fire agency regulations, but these rarely involve the extensive ecological and hazard assessments required for public land burns. Assistance from prescribed burn associations or agencies like Cal Fire is available, often covering planning and execution, including liability (McCormack et al. 2023). However, this reduced procedural rigor can increase the risk of escaped fires, especially in agricultural areas with abundant combustible materials, highlighting the need for stricter risk assessments for private land burns. Environmental factors, such as wind patterns and seasonal droughts, can further exacerbate these risks by facilitating the rapid spread of flames beyond intended boundaries (Swain et al. 2023; McCormack et al. 2023). The consequences of these escapes can be severe, impacting not only crop yields but also neighboring ecosystems and communities. Therefore, it is crucial to develop and implement enhanced management strategies and regulatory frameworks to mitigate these risks and ensure the safe implementation of prescribed fires in agricultural contexts.

The prescribed fires and escapes have similar seasonal and monthly trends in all of the above statistics, displaying two peaks close to the beginning and the end of the wildfire season. However, a reversal is observed in the months associated with their principal and secondary peaks. Prescribed fires experience a prominent peak subsequent to the summer period, while a minor peak emerges preceding summer (Fig. 1c and f). Conversely, the pattern is inverted for escaped prescribed fires (Fig. 1d and g), where the major peak occurs prior to summer and the minor peak follows the summer season. The concentrated reduction of accumulated forest fuels during these peak months can be attributed to suitable fuel moisture and climate conditions, such as temperature, humidity, and wind speed, which facilitate prescribed burning without excessive dryness and a low risk of loss of control (Chiodi et al. 2018). However, the reverse of the major and minor peaks indicates that prescribed fires executed right before or at the beginning of the wildfire seasons have a higher probability of escape and result in larger escaped areas compared to fires burned at the end of or right after the wildfire seasons. Significant periods of winter in much of California experience relative humidity, maximum air temperatures, and wind speeds that fall within the meteorological thresholds suitable for conducting prescribed fires (Baijnath-Rodino et al. 2022). These winter periods are identified as windows for effective prescribed burning under low-risk conditions, as demonstrated by previous studies (York et al. 2021; Baijnath-Rodino et al. 2022).

The general spatial pattern of escapes concentrated in northern and central California would be intuitively expected, given that more prescribed fires would be targeted to occur in regions historically adapted to a high-frequency, low-intensity fire regime. The regular implementation of crop fires on privately owned land in the Central Valley is also a primary contributor to escaped prescribed fires. In most regions of California, a consistent spatial pattern is observed where areas with higher prescribed fire activity tend to experience fewer escapes, whereas regions with higher escape frequencies typically have fewer prescribed fire activities nearby, often adjacent but non-intersecting with prescribed fire hot spots.

The spatial distribution of escaped prescribed fires across California underscores the influence of environmental conditions on fire management challenges. The identified clusters reflect various levels of impact from climates and vegetation characteristics, emphasizing the importance of customized fire management strategies. Wind speed emerges as a critical factor affecting escape likelihood, necessitating advanced predictive models to account for California’s dynamic wind patterns. The rapid and unpredictable variations in wind speed and direction during practical applications impede the comprehensive prediction of the entire wind condition profile during prescribed fires (Sanjuan et al. 2014; Sharples et al. 2012; Simpson et al. (2013). Fuel vegetation cover and specific vegetation types, such as California Montane Conifer and Ruderal Grassland, play pivotal roles in fire behavior and escape occurrences. The prevalence of escapes in areas with moderate tree cover (20−60%) highlights vulnerabilities in prescribed fire practices, particularly in regions prone to extreme weather events and dense vegetation. Agricultural lands are identified as high-risk areas for fire escapes, underscoring the need for targeted mitigation measures and enhanced monitoring protocols (Regmi et al. 2023). Integrating these findings into fire management policies can mitigate escape risks and enhance overall wildfire resilience in California’s diverse ecosystems.

## Conclusion

In this study, we investigated the temporal and spatial patterns of escaped prescribed fires from 1991 to 2020 in California. Exploring when and where the prescribed fires are more likely to escape is critical for resource managers developing forest management and fuel treatment strategies. The analyses of this study aim to reveal the seasonal and monthly trends of escape prescribed fires, their spatial distribution characteristics and structure, and the relationship between environmental variables and the occurrence of escapes.

The findings show that the implementation of prescribed fires in California has exhibited a significant upward trend since 1991, marked by a notable increase in both the number of prescribed fires and the total burned area. Similarly, the records of escaped prescribed fires also show a rising trend from 2010 to 2020. However, a substantial portion of this increase is attributed to enhanced data collection processes and improved database completeness, rather than a proportional rise in corresponding prescribed fire escapes. Seasonally and monthly, prescribed fires and escaped prescribed fires display similar patterns, with two peaks occurring close to the beginning and end of the wildfire season. Notably, the month with the highest probability of escapes does not necessarily align with the month recording the most escape incidents or the largest escape areas. In addition, prescribed fires executed before or at the start of the wildfire season are associated with a higher incidence of escape and result in larger escaped areas.

The spatial distribution of escaped prescribed fires shows cluster patterns across California, which shows that in most regions of California, areas with more prescribed fires generally experience fewer escapes, while areas with higher occurrences of escapes have less frequent prescribed fire implementations and are often adjacent to prescribed fire hot spots.

Among the observed vegetation types in escaped prescribed fires, trees with cover ranging from 20 to 60% are the most prevalent, followed by 40 to 50% herbs. Given the large coverage and complicated topography in California, the underlying causes of environmental conditions leading to escapes vary across different regions. In the central California Valley, escapes predominantly result from high temperatures and low humidity levels. Areas close to or located within the Sierra Nevada Mountains and Coastal ranges experience escape events influenced significantly by high wind speeds and abundant vegetation cover. Despite comparatively lower vegetation density in the south coast and southeast desert regions, the confluence of extremely high temperatures and wind speeds, as well as low humidity levels and precipitation, increases the risk of prescribed fire escapes.

The process of collecting information about prescribed fires revealed several challenges to the current ability to quantify the rates of prescribed fire escapes. There is no database designed specifically to capture prescribed fire escapes across landowner groups. Consequently, there is a lack of consistency in defining escapes and documenting their occurrences. Prescribed fires that occur on private land make up a significant proportion of the total number of controlled burns that occur in California. However, prescribed fires on private lands are not monitored across counties and across burn sizes consistently. If a significantly large escape event originates from a prescribed fire on private land, only then it is likely to be documented in one of the databases used in this study, biasing the true picture. However, since prescribed fires that do not escape are not monitored consistently, it is challenging to accurately estimate the rate of prescribed fire escapes. Therefore, a more systematic protocol to record both prescribed fire events and escape events uniformly across all counties will be beneficial in implementing landscape management practices. Future research should also focus on refining predictive models, expanding dataset integration, and implementing proactive fire management strategies to mitigate escape risks effectively across California’s varied landscapes.

## Supplementary Information

Supplementary Material 1.

## Data Availability

Prescribed fire and escaped prescribed fire records were retrieved from FRAP (2018), CALSTATS (2021), and MTBS U.S. Geological Survey and Nelson (2021). Environmental variables were obtained from PRISM (2020) and LANDFIRE (2016). ArcGIS Pro 2.4 ESRI (2019) and R (2021) were used to conduct spatiotemporal analysis and generate result visualizations.
