# Supplementary material for: Temporal and spatial pattern analysis of escaped prescribed fires in California from 1991 to 2020
Source: Fire Ecol. 2025 Jan 9;21(1):3. doi: 10.1186/s42408-024-00342-3 (PMC11717834; doi:10.1186/s42408-024-00342-3)
Supplement: Supplementary file 1 — Supplementary Material 1. [file 42408_2024_342_MOESM1_ESM.pdf]

Supplementary Information for "Temporal and spatial pattern analysis of escaped prescribed fires in California from 1991 to 2020"

Shu Li[1], Janine A. Baijnath-Rodino[1], Robert A. York[2], Lenya N. Quinn-Davidson[3], Tirtha Banerjee[1]

[1]Department of Civil and Environmental Engineering, University of California, Irvine, Irvine, CA, USA

[2]Department of Environmental Science, Policy, and Management, University of California, Berkeley, Berkeley, CA, USA

[3]University of California Agriculture and Natural Resources, Eureka, CA, USA

Contents of this file

1. Data - Environmental Variables

2. Methods - Outliers Detection and Treatment

3. Methods - Bayesian Models

4. Methods - Complete Spatial Randomness Test

5. Methods - Identification of Clustering or Repulsion Patterns using G, K and L Function

6. Methods - Kernel Density Estimation

7. Methods - Logistic Regression

8. Results - Spatiotemporal Patterns for prescribed fires and escaped prescribed fires excluding agricultural fires

1. Data - Environmental Variables

Fig. S1 (a)-(c) shows the annual 30-year normal of precipitation, maximum VPD, and maximum temperature. Topography data of elevation, slope, and aspect were also extracted from PRISM (Fig. S1 (d)-(f)). The 30-m resolution of Fuel Vegetation Cover (FVC) and national vegetation classification are shown as Fig. S1 (g)&(h).

2. Methods - Outliers Detection and Treatment

The temporal distributions of prescribed fires and escaped prescribed fires, including those with burned areas exceeding 5,000 acres, are shown in Fig. S2. In panel (b), notably, the years 2000, 2006, and 2009 exhibit significant peaks in the burned area of escaped prescribed fires compared to other years. Similarly, the months of February, August, and September show markedly higher total burned areas over the past three decades. However, these peaks do not indicate a consistent temporal trend, as they result from single, extremely large escaped prescribed fires. To accurately depict the general trends in the yearly and monthly distribution of prescribed fire occurrences and burned areas, we excluded these extreme cases from the main analyses.

3. Methods - Bayesian Models

The framework of the Binomial-Beta model is outlined in equations (1) - (4), with abbreviations Bin, Be and LN representing the Binomial, Beta and Log-normal distribution respectively. Equation (1) was applied to describe the posterior distribution of the occurrence probability of escaped prescribed fires in each year and month taking the prior information, that is, the historical escape records in the same region into account. y_{ij} represents escape event records each year and each month, j denotes distinct months, θ represents the escape probability and n represents the total count of prescribed fires. Given the limited prior information regarding escaped prescribed fires, we adopted uninformative priors to provide a vague initial assessment of escape occurrences, as shown

in equation (2). The parameters α and β in the Beta distribution were used to characterize the distribution of θ, signifying counts of escapes and non-escapes in all prescribed fires. In this context, μ represents the escape ratio, while η represents the total count of prescribed fires (equation (3)). Thus hyper-parameters a, b, m, and c were applied to delineate μ and η using Beta distribution and Log-normal distribution (equation (4)).

$$y_{ij}|\theta_j \sim Bin(n_j, \theta_j) \tag{1}$$

$$\theta_j|\alpha, \beta \sim Be(\alpha, \beta) \tag{2}$$

$$\mu = \frac{\alpha}{\alpha + \beta}, \quad \eta = \alpha + \beta \tag{3}$$

$$\mu \sim Be(a, b), \quad \eta \sim LN(m, c) \tag{4}$$

Similarly, the structure of Poisson-Gamma model is shown in equations (5) - (8), where θ represents the occurrence number of escaped prescribed fires each month, μ represents the mean counts and m represents the mode of counts. Equation (5) describes the posterior information of occurrence counts of escaped prescribed fires each month, considering the historical monthly counts described in equation (6). Equation (7) and (8) chose the mean counts μ and the mode m as hyper-parameters to decide the shape and scale parameter α and β in the prior information.

$$y_j|\theta_j \sim Pois(\theta_j) \tag{5}$$

$$\theta_j|\alpha, \beta \sim Gamma(\alpha, \beta) \tag{6}$$

$$\alpha = \frac{\mu + 1}{\mu + m}, \quad \beta = \frac{1}{\mu + m} \tag{7}$$

$$\mu \sim LN(m, c), \quad \beta \sim Be(a, b) \tag{8}$$

4. Methods - Complete spatial randomness test

In this test, California was divided into 6×10 grids (Fig. S3(b))., with the count of escapes in each unit recorded. The intensity of escaped prescribed fires was subsequently calculated by determining the number of points within each grid, estimated from the density of observations. Through the application of the Monte Carlo method, this test simulated 99 random point processes across California, calculating the χ^2 statistic for both observed events and simulations. The χ^2 (chi-squared) statistic serves as a metric of the variation between observed and expected point distributions in the absence of any relationship between them. The resulting p-value can then be determined by ranking the test statistics of the observed point process. The equation for χ^2 statistics is shown below:

$$\chi^2 = \sum \frac{(O_i - E_i)^2}{E_i} \tag{9}$$

Where O_i is the observed value and E_i is the expected value. The p-value would be obtained based on the ranking of the test statistics of the observed point process.

5. Methods - Identification of clustering or repulsion patterns using G, K and L function

The G function measures the distribution of distances from an arbitrary point to its nearest neighbor:

$$G(r) = \frac{N(d_i \leq r)}{n} \tag{10}$$

where d_i is the nearest neighbor distances from a random event to its nearest event, and n is the total number of events. The theoretical distribution of G function is $G_{pois}(r) = 1 - exp(-\lambda \pi r^2)$, and the estimated value of G(r), represented by $\hat{G}(r)$, larger than $G_{pois}(r)$ suggests a clustered pattern.

The K function, proposed by Ripley (Ripley, 1977), in addition, takes multiple pairwise distances into account. Then the empirical distribution function of pairwise distances is weighted and normalized:

$$K(r) = \frac{E(N(b(\mathbf{0}; r)))}{\lambda} \tag{11}$$

where $N(b(\mathbf{0}; r))$ means the number of events in a sphere with radius r and centered at the origin of study region, and E represents the expectation. $K_{pois}(r) = \pi r^2$ and the estimated K(r) larger than $K_{pois}(r)$ suggests a clustered pattern.

Besag (Besag, 1977) proposed the L-function to remove the effect of the volume of the sphere and transform the Poisson K function into a straight line:

$$L(r) = \sqrt{\frac{K(r)}{\pi}}, \tag{12}$$

where $L_{pois}(r) = r$, and the estimated $L(r)$ larger than $L_{pois}(r)$ suggests a clustered pattern.

As shown in Fig.S4, the estimated functions based on escaped prescribed fire records (observations) consistently exceed the theoretical distribution, indicating that the observed point patterns have more points than expected in the theoretical homogeneous Poisson distribution. This suggests that the spatial distribution of the escaped prescribed fires is clustered.

6. Methods - Kernel Density Estimation

The equations of kernel density estimation were as follows:

$$\widehat{\lambda_h}(x) = \sum_{i=1}^{n} \frac{K_h(x - x_i)}{C_h(x_i)} \tag{13}$$

$$K_h(x - x_i) = exp(-(\frac{|x - x_i|}{h})^2) \tag{14}$$

$$C_h(x) = \int K_h(x - x_i)dx \tag{15}$$

Where $x_i \in \{x_1, x_2, ..., x_n\}$ represents observed points in the study region, $K_h(x - x_i)$ is a kernel function with bandwidth h, $C_h(x)$ is an edge correction factor for missing observations caused by edge effects. The inclusion of edge effects in the KDE estimation is due to the fact that escapes occurring outside of California's administrative boundary may still interact with escapes occurring within California.

7. Methods - Logistic Regression

The logistic regression equation is as follows:

$$ln(\frac{P}{1 - P}) = w_0 + w_1x_1 + ... + w_nx_n \tag{16}$$

Here, P represents the probability of escape occurrence, x denotes various environmental characteristics, and w signifies the corresponding weights of x.

8. Results - Spatiotemproal Patterns for prescribed fires and escaped prescribed fires excluding agricultural fires

The temporal and spatial statistics for prescribed fires and escaped prescribed fires, excluding agricultural use fires, are presented below. The inclusion or exclusion of agricultural fires does not significantly impact the spatial or temporal patterns(Fig. S5, Fig. S6). The contribution of environmental variables to escapes remains the same, as the coefficients and p-values for each environmental variable in the logistic regressions remain unchanged. The only notable change occurs in the dominant vegetation types and land uses associated with escaped prescribed fires (Table S1). With agricultural fires excluded,

developed roads no longer appear as a dominant land use type. Instead, areas with moderate-density tree, shrub, and herb cover become the primary land use types where escapes are most frequent.

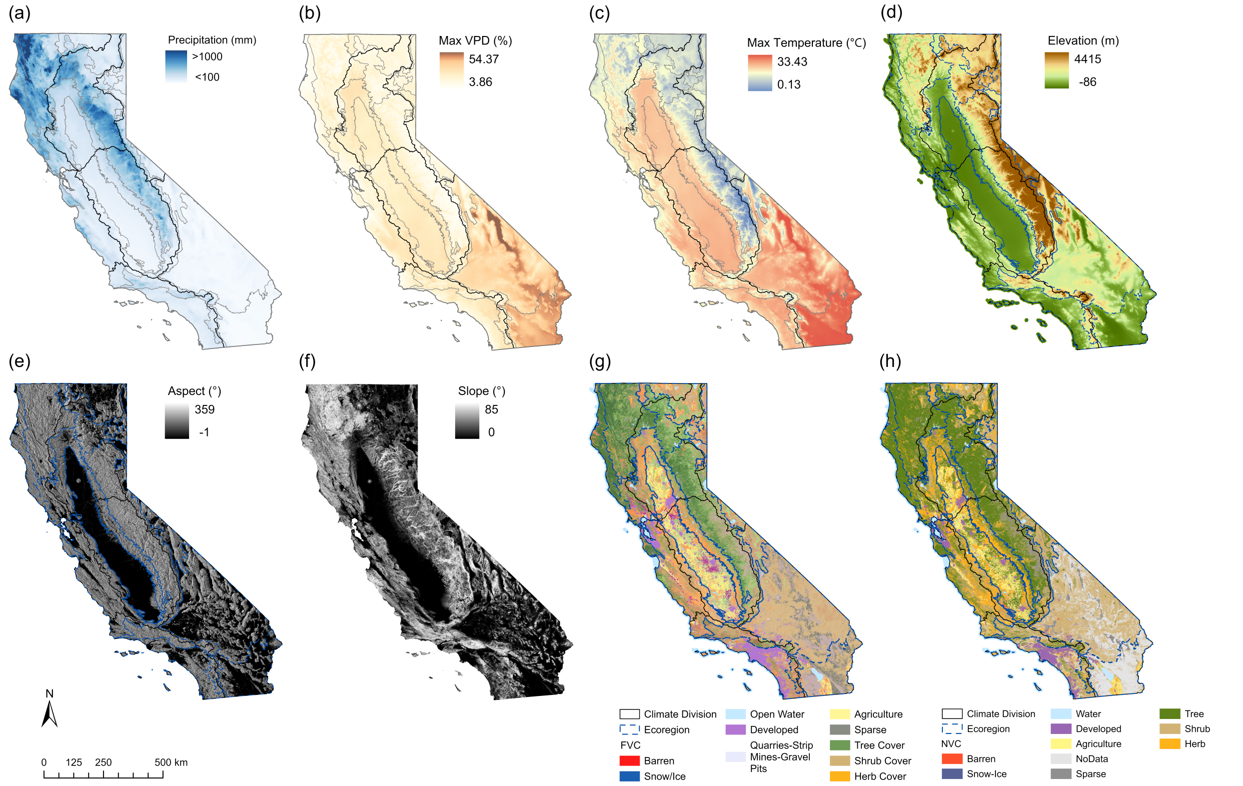

Figure S1. Environmental Factors Potentially Influencing Escaped Rx Fires. Panels (a) to (c) represent the 30-year normal annual precipitation, the maximum vapor pressure deficit, and the maximum temperature, respectively, obtained from the PRISM dataset. Panels (d) to (f) illustrate elevation, aspect and slope from the PRISM dataset. Panels (g) and (h) show fuel vegetation cover and national vegetation classification, respectively, obtained from the LANDFIRE dataset.

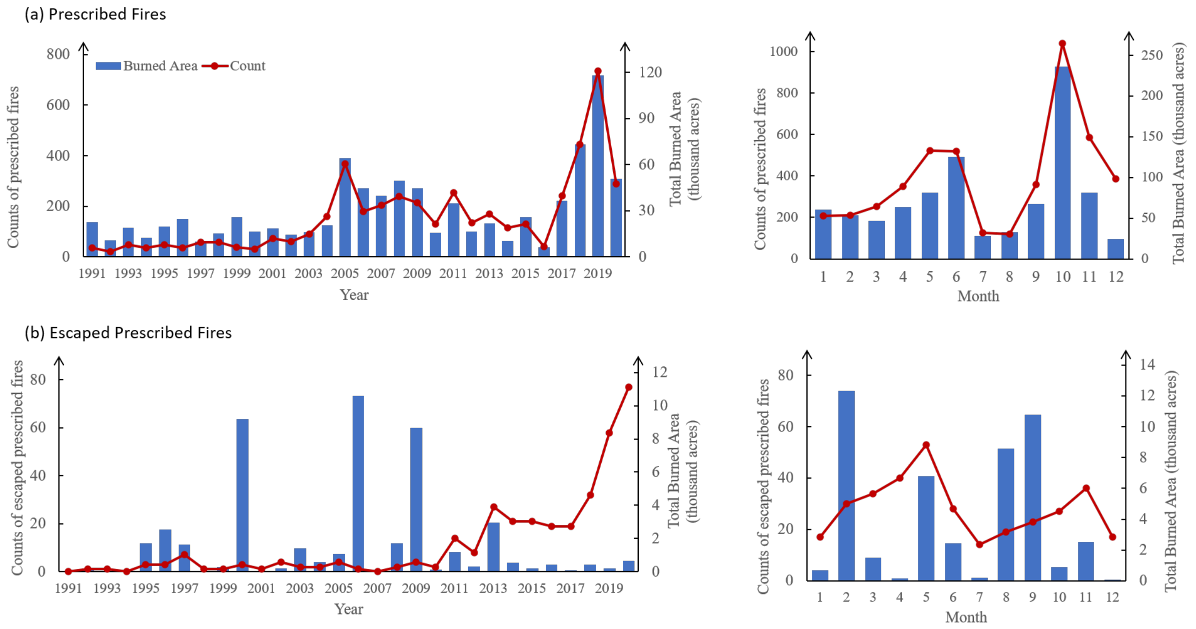

Figure S2. Temporal Patterns of Rx Fires and Escaped Rx Fires in California from 1991 to 2020. Temporal patterns are depicted on the basis of yearly and monthly occurrences. The red points and lines correspond to fire counts, the blue columns indicate the total burned area of the fires

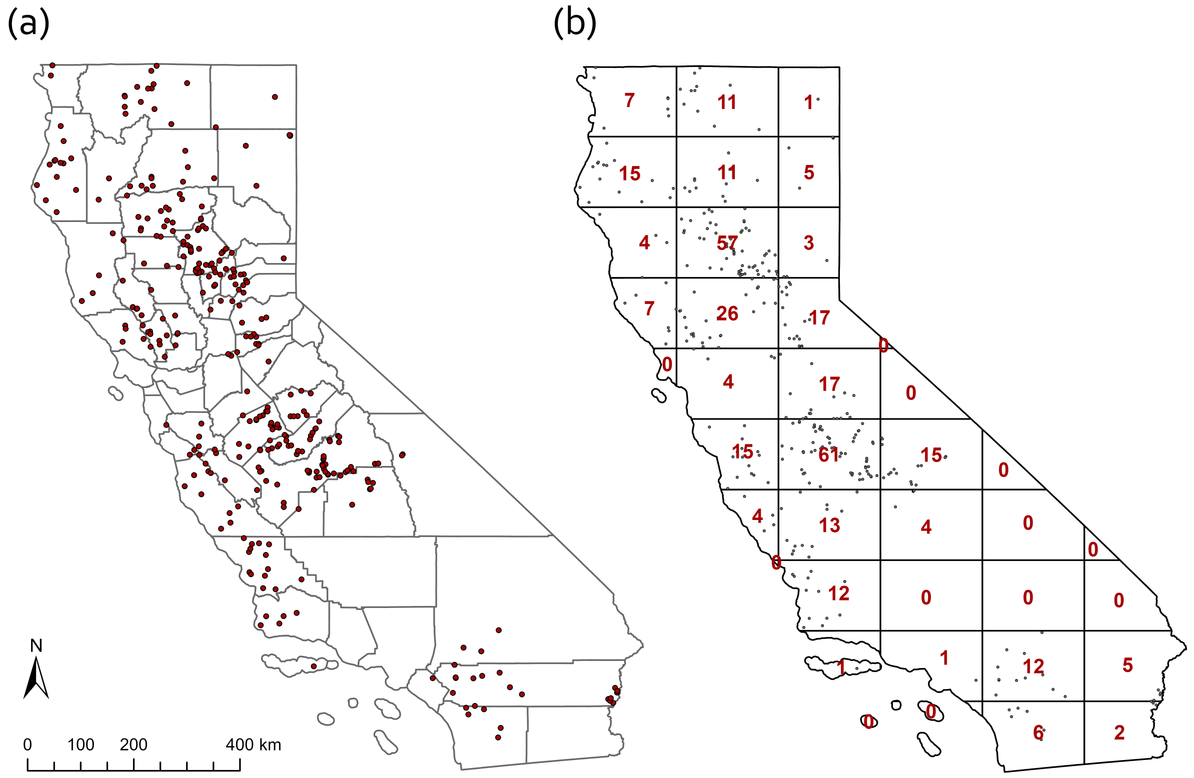

Figure S3. Spatial Distribution of Escaped Rx Fires in California from 1991 to 2020. Panel (a) shows the locations of escaped Rx fires in county boundaries; (b) shows the escaped Rx Fire Counts in Quadrats, dividing California into 6 columns and 10 rows, for the Complete Spatial Randomness (CSR) Test.

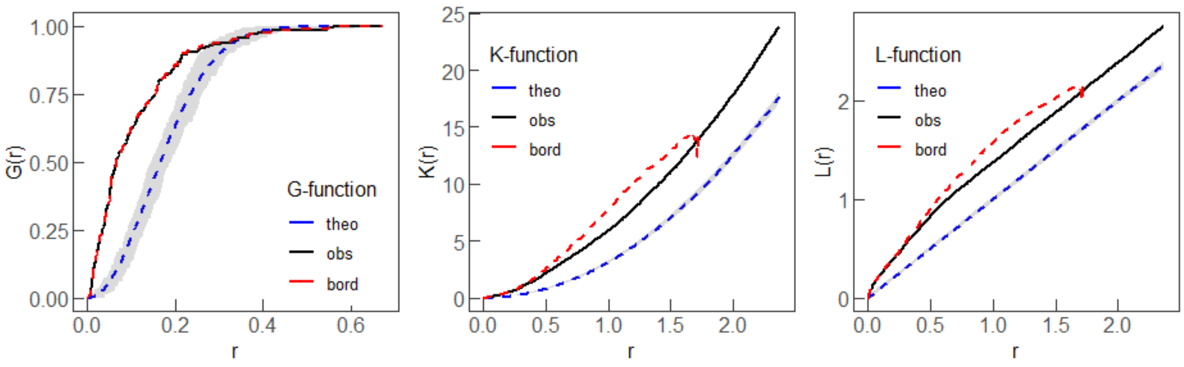

Figure S4. G, K, L estimation for the occurrence of escaped prescribed fires in California from 1991 to 2020. r denotes the distance between points, which is the radius in the functions. The blue dash line (theo) represents the theoretical Poisson distribution, the black line (obs) represents the empirical distribution of escaped prescribed fires, the red dash line (bord) represents the edge-corrected empirical distribution with border correction, the gray envelope represents the highest and lowest possible range of theoretical Poisson distribution.

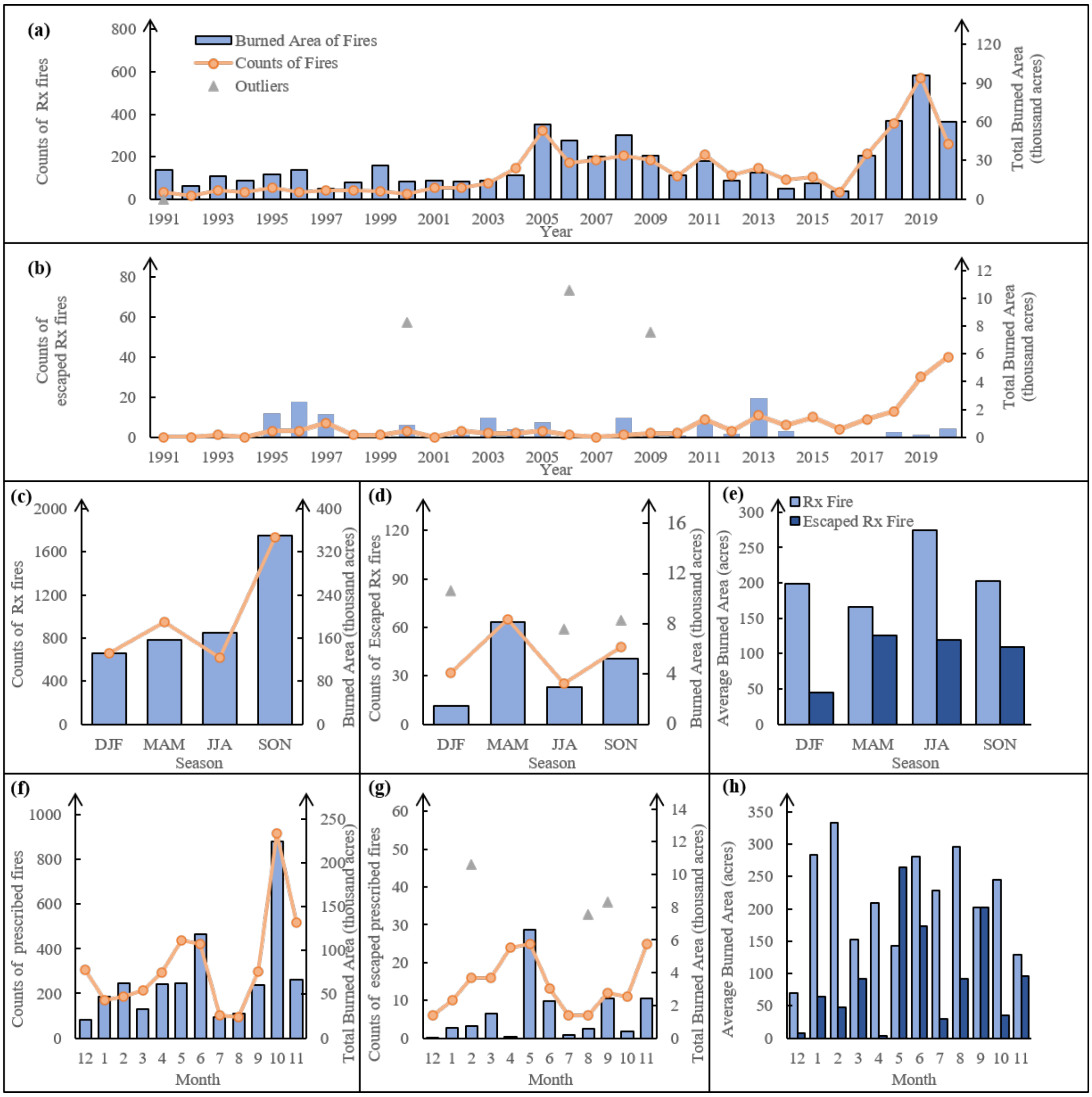

Figure S5. Temporal Patterns of Rx Fires and Escaped Rx Fires without agricultural fires in California from 1991 to 2020. Temporal patterns are depicted on the basis of yearly (a), (b)), seasonal ((c)-(e)), and monthly ((f)-(h)) occurrences. The seasons are denoted by the months they contain, with DJF representing winter, MAM representing spring, JJA representing summer, and SON representing autumn. Panels (a), (c), and (f) present data for Rx fires, while panels (b), (d), and (g) represent escaped Rx fires. Panels (e) and (h) represent the average burned area for a single Rx fire and a single escaped Rx fire in each season ((e)) and each month ((h)). The orange points and lines correspond to fire counts, the blue columns indicate the total burned area of the fires, and the gray triangles represent the outliers in escaped prescribed fires with burned areas larger than 5,000 acres (2023 hectares).

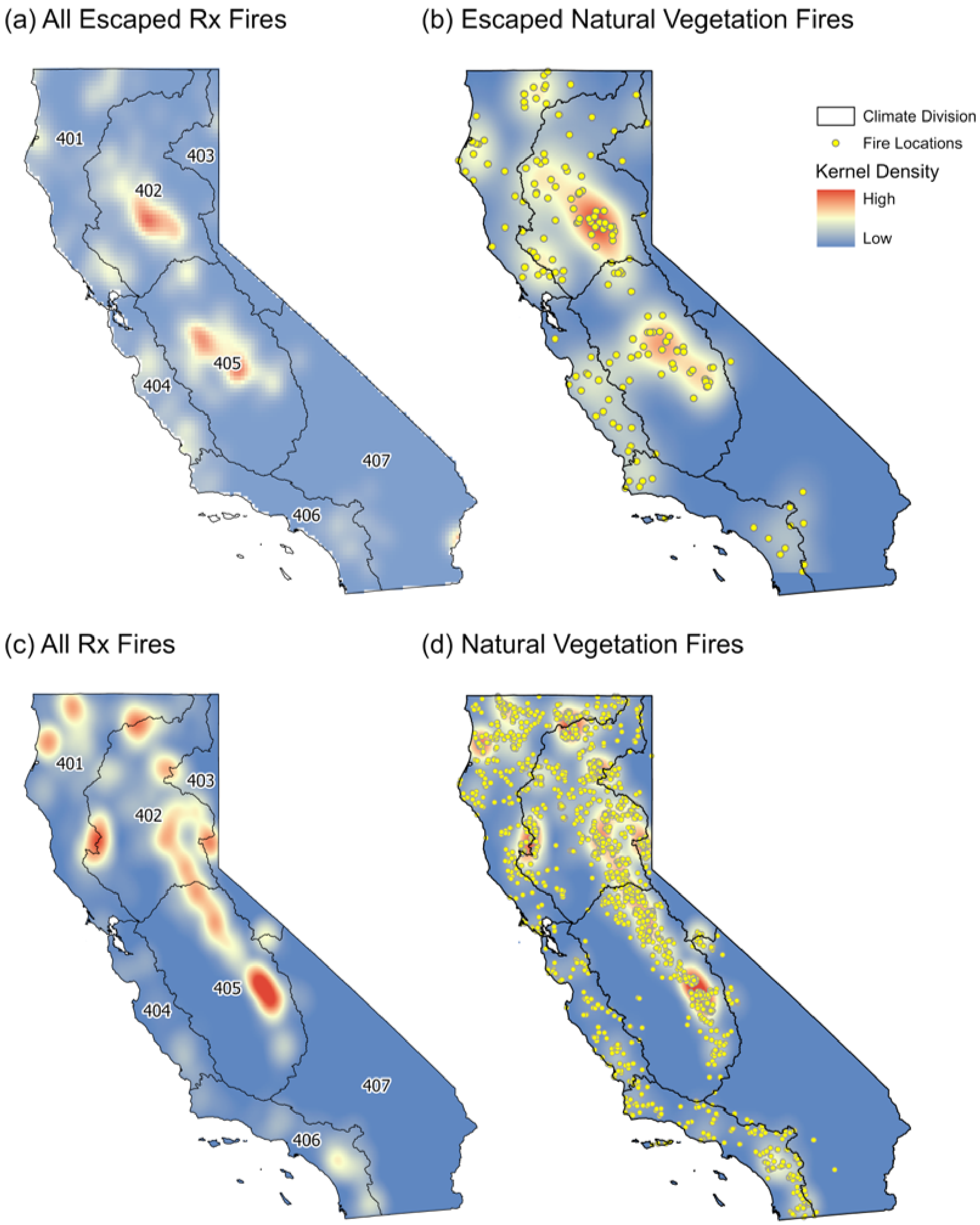

Figure S6. Estimation of kernel density for (a) all escaped prescribed fires, (b) escaped natural vegetation fires, (c) all prescribed fires, (d) prescribed natural vegetation fires

Vegetation Type	Percentage (%)	Accumulated Percentage (%)
**Fuel Vegetation Cover**		
$40 \leq$ Tree Cover $< 50\%$	17.34	17.34
$50 \leq$ Tree Cover $< 60\%$	9.83	27.17
$40 \leq$ Shrub Cover $< 50\%$	8.67	35.84
$30 \leq$ Tree Cover $< 40\%$	7.51	43.35
$40 \leq$ Herb Cover $< 50\%$	7.51	50.86
**National Vegetation Classification**		
Californian Montane Conifer Forest & Woodland	16.76	16.76
California Xeric Chaparral	13.87	30.63
California Broadleaf Forest & Woodland	13.29	43.92
California Ruderal Grassland & Forb Meadow	11.56	55.48

Table S1. Dominant Vegetation Classes and Land Use Type in Escaped Natural Vegetation Fires. The table displays the highest-ranking vegetation species until the cumulative percentage reaches 50%.

References

Besag, J. (1977). Comments on ripley's paper: Royal statistical society. *Journal*, *39*, 193–195.

Ripley, B. D. (1977). Modelling spatial patterns. *Journal of the Royal Statistical Society: Series B (Methodological)*, *39*(2), 172–192.
